# Paired TIR-NLRs of *Arabidopsis thaliana* have diverse recognition and activation mechanisms

**DOI:** 10.3389/fpls.2026.1958275

**Published:** 2026-10-19

**Authors:** Hayden Burdett, Tana L. Holmes, Hee-Kyung Ahn

**Affiliations:** Institute of Molecular Plant Sciences, Daniel Rutherford Building, Kings Buildings, University of Edinburgh, Edinburgh, United Kingdom

**Keywords:** Arabidopsis, Brassicaceae, paired NLR, plant immunity, TIR, TIR-NLR

## Abstract

Recognition of pathogens by immune receptors is essential for immune response activation in plants. The NLR (Nucleotide-binding, Leucine-rich repeat) family of intracellular immune receptors recognise the presence of pathogens from within host plant cells. Some NLRs are exclusively ‘paired,’ and require each other for function. With the pathogen sensing and immune activation function separated between the ‘sensor’ and the ‘executor’ NLR, the paired NLR system is promising target for engineering for broad spectrum disease resistance. Here, we review our current understanding of the diversity, molecular mechanism, and evolution of Arabidopsis paired NLRs with N-terminal TIR (Toll, Interleukin-1 receptor) domains (TIR-NLRs). We conclude with examples of paired TIR-NLRs beyond Arabidopsis and Brassicaceae species.

## Introduction

Immune receptors of all life forms enable rapid, sensitive, and specific detection of pathogens, followed by activation of immune signalling. In plants, this recognition occurs in two different locations, at the cell surface by PRRs (Pattern Recognition Receptors), and within the cell by NLRs (Nucleotide-binding, Leucine-rich repeat Receptors) (Jones et al., 2024). Recognition of molecular patterns at the cell surface induces PTI (pattern triggered immunity) and within the cell, NLRs recognise pathogen effectors secreted into the host, inducing ETI (effector-triggered immunity) (Locci and Parker, 2024). Most NLRs can function as singletons to recognise pathogen effector and induce immune signalling, culminating in a localised cell death response known as HR (Hypersensitive Response). However, other NLRs can also work together with another NLR, often exclusively as pairs (Xi et al., 2022) or redundantly as networks (Adachi and Kamoun, 2022).

Upon effector recognition, NLRs oligomerise or undergo conformational changes that induce proximity of their signalling domains (Kim et al., 2026). NLRs are classified depending on their N-terminal signalling domain. NLRs with CC (Coiled-Coil) domain (CNLs) oligomerise and undergo conformational change that induces Ca^2+^ influx by channel formation upon sensing a pathogen (Bi et al., 2021). TIR domains from multiple kingdoms exhibit NADase activity upon oligomerization, and this is the main function of NLRs with TIR domains (TNLs) in plants (Wani and Pukkila-Worley, 2025). TIR domains cleave nicotinamide from NAD^+^ (Nicotinamide adenine dinucleotide) (Horsefield et al., 2019; Wan et al., 2019) to produce small molecules involved in immune signalling including di-ADPR (di-adenosine diphosphate ribose), ADPr-ATP (Jia et al., 2022), 2’cADPR (Manik et al., 2022), and pRib-AMP/ADP (Huang et al., 2022) (pRibose-AMP/ADP). Di-ADPR and ADPr-ATP interact with the EDS1 (Enhanced Disease Resistance 1)/SAG101/Senescence-Associated Gene 101) signalling module, whilst pRib-AMP/ADP interacts with the EDS1/PAD4 (Phytoalexin Deficient 4) signalling module (Huang et al., 2022; Jia et al., 2022). Binding of these small molecules to the EDS1 family of proteins activates a third class of NLRs, the CC_RPW8_-NLRs, (RESISTANCE TO POWDERY MILDEW 8) (RNLs) (Xi et al., 2022). In Arabidopsis, EDS1/SAG101 with RNL NRG1 (N-Requirement Gene 1) contribute to host cell death, whilst EDS1/PAD4 with RNL ADR1 (Activated Disease Resistance 1) mainly drives pathogen growth restriction (Lapin et al., 2020). In Solanaceae, a group of NLRs in the NRC (NLR Required for Cell death) clade form ‘sensor/helper’ networks (Adachi and Kamoun, 2022). Both TNLs and CNLs can be found to function together as ‘pairs’, with RPS4 (Resistance to *Pseudomonas syringae* 4)/RRS1 (Resistance to *Ralstonia solanacearum* 1) TNL pair in Arabidopsis, and RGA4/RGA5 CNL pair in rice among the most extensively studied examples (Xi et al., 2022).

*NLR* genes are responsible for heritable resistance traits against many commercially relevant crop pathogens, and are an effective form of durable, environmentally sustainable resistance, particularly when used in NLR ‘stacks’ (Luo et al., 2026), applying multiple selection pressures on pathogens to prevent them evolving resistance against a single *NLR* gene. Recent studies highlighted the strong potential for NLR engineering in novel disease resistance (Xi et al., 2026; Zhu et al., 2026). However, clear understanding of the molecular mechanisms by which effector recognition and NLR activation are regulated is still required to guide precise editing of NLRs. Some paired CNLs have been engineered for expanded recognition of multiple effectors through precise structure guided edits to their IDs (Integrated Domains), which are non-canonical domains integrated into NLRs involved in pathogen effector sensing (De La Concepcion et al., 2019; Liu et al., 2021; Kourelis et al., 2023). CNL pairs and TNL pairs have differing downstream signalling requirements for disease resistance, and therefore TNL pair engineering will broaden resistance pathways available to a plant host. This, combined with an additional pool of IDs for engineering, makes TNL pairs an attractive prospect for the further layering of robust resistances to prevent pathogen evolution and overcoming single *NLR* gene-mediated resistance.

In this review, we discuss characteristics and mechanics of paired TNLs before and after activation, their co-evolution, and distribution across species. Focussed reviews on paired CNLs, or of NLR networks can be found in other reviews (Adachi and Kamoun, 2022; Xi et al., 2022).

## Classification - what are paired TNLs?

Some NLRs need other NLR(s) for function (Van Wersch and Li, 2019). Paired TNLs comprise both a ‘sensor’ NLR, often including ID(s), which recognises the effector and an ‘executor’ NLR, that is required for immune signalling activation. In addition to ID(s), sensor paired NLRs exhibit diverged sequence features compared to executor paired NLRs or singleton NLRs (see below section). Paired NLRs often share a promoter and are divergently transcribed (**Figure 1A**). An exception is the tandemly oriented RPP2A (Resistance to *Peronospora parasitica* 2A) and RPP2B paired TNL, that confers resistance against Hpa (*Hyaloperonospora arabidopsidis*) (Sinapidou et al., 2004). In Arabidopsis, around 15 genomically juxtaposed NLR pairs can be classified as paired NLRs (Van De Weyer et al., 2019) (**Figure 1B**; **Table 1**). All Arabidopsis paired NLRs are TNLs and form two distinct clades based on the NB (Nucleotide-Binding) domain phylogeny (Van De Weyer et al., 2019). The two clades are classified as RPS4-like and SOC3 (Suppressor of *chs1-2*, 3)-like (Van De Weyer et al., 2019), or clade A and B, respectively (Lee and Chae, 2020). Each clade is further divided into two subclades, with the sensor and executor pairs of these clades clustering together, forming distinct subclades of sensor and executors of RPS4-like and SOC3-like clades, respectively (Van De Weyer et al., 2019). For both clades, autoactivity of the executor is repressed by co-expression of the sensor (Castel et al., 2019). The sensors of RPS4-like clades have various IDs, whereas the sensors of SOC3-like clades consist exclusively of genes containing only TIR and NB-ARC domains, also known as TNs (TIR-NBs) (**Figure 1C**).

**Figure 1:**
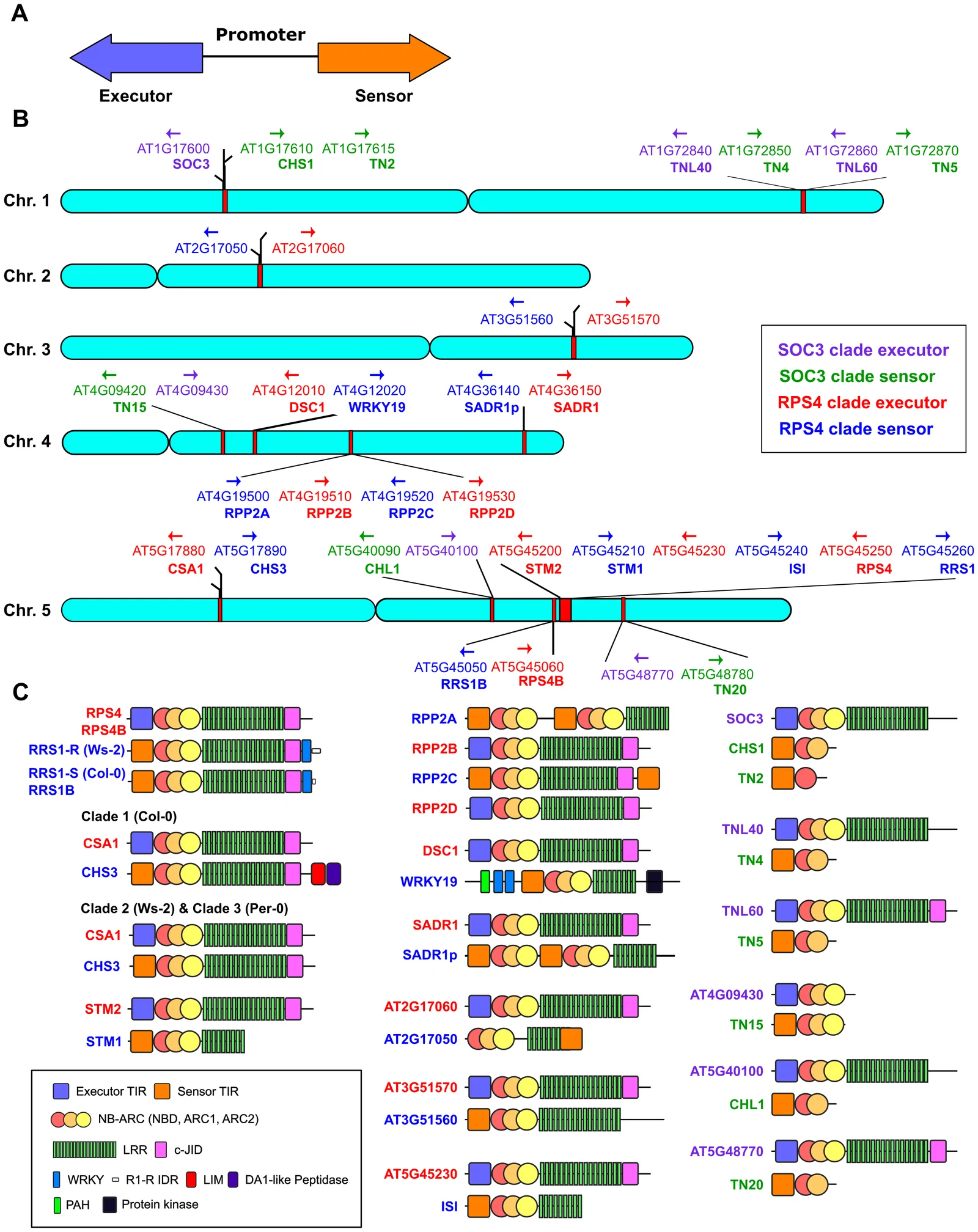
Genomic positions and domain architecture of paired NLRs in Arabidopsis Col-0. **(A)** General genome architecture of paired NLRs. **(B)** Genomic positions of paired NLRs in Arabidopsis. Arrow indicates direction of transcription, and colour indicates executor/sensor and clade (SOC3 executor, purple; SOC3 sensor, green; RPS4 executor, red; RPS4 sensor blue). AGI locus, and gene name (when present) are labelled. **(C)** Domain architecture of paired NLRs. Domain positions of paired NLRs are shown based on predictions from TAIR (www.arabidopsis.org). Different symbols and/or colours are used for the different domains. TIR, Toll/Interleukin-1 receptor; NB-ARC, Nucleotide Binding domain shared by APAF-1, apoptotic protease-activating factor 1; R proteins and CED-4 ,cell death protein 4; LRR, Leucine Rich Repeat; c-JID, C-terminal jelly roll/Ig-like domain; WRKY, WRKY transcription factor domain; R1-R IDR, RRS1-R Intrinsically Disordered Region; LIM, Lin-11, Isl-1, and Mec-3 domain; PAH, paired amphipathic helix.

**Table 1:** Paired NLRs from Arabidopsis Col-0.

Clade	AGI locus	Other name(s)	Strand	Sensor/executor	First appearance of name
SOC3	AT1G17600	SOC3, WRR12	–	Executor	Zhang et al. (2017b) PMID: 27699788
					Cevik et al. (2019) PMID: 30692254
SOC3	AT1G17610	CHS1	+	Sensor	Wang et al. (2013) PMID:23651299
					Zbierzak et al. (2013) PMID:23617639
SOC3	AT1G17615	TN2	+	Sensor	Zhao et al. (2015) PMID:25617755
SOC3	AT1G72840	TNL40	–	Executor	Chen et al. (2021) PMID:33924478
SOC3	AT1G72850	TN4	+	Sensor	
SOC3	AT1G72860	TNL60	–	Executor	Chen et al. (2021) PMID:33924478
SOC3	AT1G72870	TN5	+	Sensor	
SOC3	AT4G09420	TN15	–	Sensor	
SOC3	AT4G09430		+	Executor	
SOC3	AT5G40090	CHS1-LIKE 1 (CHL1), TN18	–	Sensor	Zbierzak et al. (2013) PMID:23617639
SOC3	AT5G40100		+	Executor	
SOC3	AT5G48770		–	Executor	
SOC3	AT5G48780	TN20	+	Sensor	
RPS4	AT2G17050		–	Sensor	
RPS4	AT2G17060		+	Executor	
RPS4	AT3G51560		–	Sensor	
RPS4	AT3G51570		+	Executor	
RPS4	AT4G12010	DSC1	–	Executor	Lolle et al. (2017) PMID:28407487
					Warmerdam et al. (2020) PMID:32054439
RPS4	AT4G12020	WRKY19	+	Sensor	Warmerdam et al. (2020) PMID:32054439
RPS4	AT4G19500	RPP2A	+	Sensor	Sinapidou et al. (2004) PMID:15165183
RPS4	AT4G19510	RPP2B	+	Executor	Sinapidou et al. (2004) PMID:15165183
RPS4	AT4G19520	RPP2C	–	Sensor	Kim et al. (2024) PMID:38742296
RPS4	AT4G19530	RPP2D	+	Executor	Kim et al. (2024) PMID:38742296
RPS4	AT4G36140	SADR1	–		Jacob et al. (2023) PMID:36893276
RPS4	AT4G36150	SADR1p	+		Jacob et al. (2023) PMID:36893276
RPS4	AT5G17880	CSA1	–	Executor	Faigón-Soverna et al. (2006) PMID:17114357
RPS4	AT5G17890	CHS3	+	Sensor	Bi et al (2021) PMID:22639607
RPS4	AT5G45050	RRS1B	–	Sensor	Saucet et al. (2015) PMID:25744164
RPS4	AT5G45060	RPS4B	+	Executor	Saucet et al. (2015) PMID:25744164
RPS4	AT5G45200	STM2	–	Executor	Gao et al. (2026) PMID:42092167
RPS4	AT5G45210	STM1	+	Sensor	Gao et al. (2026) PMID:42092167
RPS4	AT5G45230		–	Executor	
RPS4	AT5G45240	ISI	+	Sensor	Dunken et al. (2024) PMID:39603244
RPS4	AT5G45250	RPS4	–	Executor	Gassmann et al. (1999) PMID:10571887
RPS4	AT5G45260	RRS1	+	Sensor	Deslandes et al. (2002) PMID:11842188
Unpaired	AT4G16930	SNC1	–		Bi et al. (2021) PMID:14576290
Unpaired	AT4G16940	SIKIC1	–		Dong et al. (2018) PMID:30082764
Unpaired	AT4G16950	SIKIC2	–		Dong et al. (2018) PMID:30082764
Unpaired	AT4G16960	SIKIC3	–		Dong et al. (2018) PMID:30082764
Unpaired	AT3G04210	TN13	–		Roth et al. (2017) PMID:28901644
Unpaired	AT3G04220	TNT1	–		Lüdke et al. (2025) BioRxiV

List of paired TNLs identified in Arabidopsis Col-0 with its clade (SOC3 or RPS4), type (sensor or executor), and study where the gene has first appeared is shown. Group of ‘Unpaired’ NLRs are also listed that may act cooperatively.

The exclusive requirement of one another for function distinguishes paired NLRs from sensor/helper NLRs. CHS3 (CHilling-Sensitive 3)/CSA1 (Constitutive Shade Avoidance 1) can be divided into three clades from sequence variations across 64 Arabidopsis natural accessions (Van De Weyer et al., 2019). Clade 1 CHS3 alleles contain LIM (Lin-11, Isl-1, and Mec-3)-peptidase (also known as DA1-like) domains integrated into the protein (Gu et al., 2023), whereas clade 2 and clade 3 CHS3 proteins do not have IDs (Yang et al., 2022). Even though sequence similarity of CSA1 is high among the three clades, and interclade interactions are observed, these interclade combinations are non-functional (Yang et al., 2022). Despite the coexistence of RPS4/RRS1 and its duplicated pair RPS4B/RRS1B in most Arabidopsis accessions, these pairs also cannot function as chimeric pairs (Saucet et al., 2015). Whilst RRS1-R chimera with TIR domain from RRS1B is able to recognise effectors, swaps of other domains fail to recognise the cognate effectors, or lead to autoimmunity (Ma et al., 2018). This suggests co-evolution of the components of each pair to strictly interact and function with the specific pair partner.

Constitutive and strong interaction between the sensor and the executor is another characteristic of paired TNLs. RPS4 and RRS1 interact readily in Arabidopsis (Ngou et al., 2020; Ahn et al., 2025), as well as in heterologous *N. benthamiana* transient expression system (Williams et al., 2014; Huh et al., 2017). Similar interaction has also been observed for CHS3/CSA1 (Yang et al., 2024) and the recently characterised Arabidopsis TNL pair STM2/STM1 (Sensitive to Transition Metals) (Gao et al., 2026). Interestingly, all the above mentioned paired TNLs maintain strong interaction in the absence of effector recognition or activation (Huh et al., 2017; Yang et al., 2024; Gao et al., 2026). This is in contrast to the transient nature of NRC sensor/helper NLRs where only a weak association is detected with proximity labelling in the absence of effector (Huang et al., 2023; Szymansky, 2025), and only NRC helper NLRs form active oligomers for immune signalling (Liu et al., 2024; Madhuprakash et al., 2024). The strong interaction of sensor/executor pair is consistent with the role of sensor TIR-pair in repressing the executor TIR-pair in the absence of activation (see below).

### Sequence variation of paired sensor NLRs

Sensor NLRs diverged from executor and canonical singleton NLRs in a multitude of ways. NB domain phylogeny-based definition of TNL class A in Meyers et al. (2003) captures TNL sensor NLRs (Meyers et al., 2003). They can contain IDs for effector recognition and inhibition of the executor, and their TIR domain(s) have lost the ability to hydrolyse NAD^+^ and produce secondary metabolites (Bayless et al., 2025). Some sensor NLRs have truncated LRR domains, and the c-JID (C-terminal jelly roll/Ig-like domain)/PL (Post LRR) domain is also diverse between sensor pairs (Saucet et al., 2021). Using Arabidopsis pairs, we examine how sensors are divergent from executor NLRs.

### TIR domains

As mentioned above, plant TIR domains are enzymes capable of producing small molecules derived from NAD^+^ and nucleic acids. For detailed reviews on the function of plant TIR domains, see Maruta et al. (2026b) (Maruta et al., 2026b). There are numerous well characterised and conserved features in the enzymatic TIR domains (**Figure 2A**), including the AE interface (Williams et al., 2014), BB-loop (Horsefield et al., 2019; Ma et al., 2020; Martin et al., 2020), catalytic glutamate (Horsefield et al., 2019; Wan et al., 2019) on the αC helix, triple αD helix arrangement at the DE surface (Chan et al., 2010; Bernoux et al., 2011), and the FRGxxxR motif in the AA loop region (Burdett et al., 2021). The first sensor NLR TIR domain to be characterised structurally was that of the RRS1. It differs from the canonical enzymatic plant TIR domain (Horsefield et al., 2019; Wan et al., 2019), with RRS1 missing the catalytic glutamate and containing only a single αD helix (Williams et al., 2014) which is more synonymous with scaffold mammalian TIR domains (Nimma et al., 2021; Kobe et al., 2025). All sensor NLR TIR domains from Col-0 Arabidopsis characterised and predicted by genomic positioning have lost some aspect of the NAD^+^ binding and small molecule synthesis sites, compromising their ability for these functions (Bayless et al., 2025).

**Figure 2:**
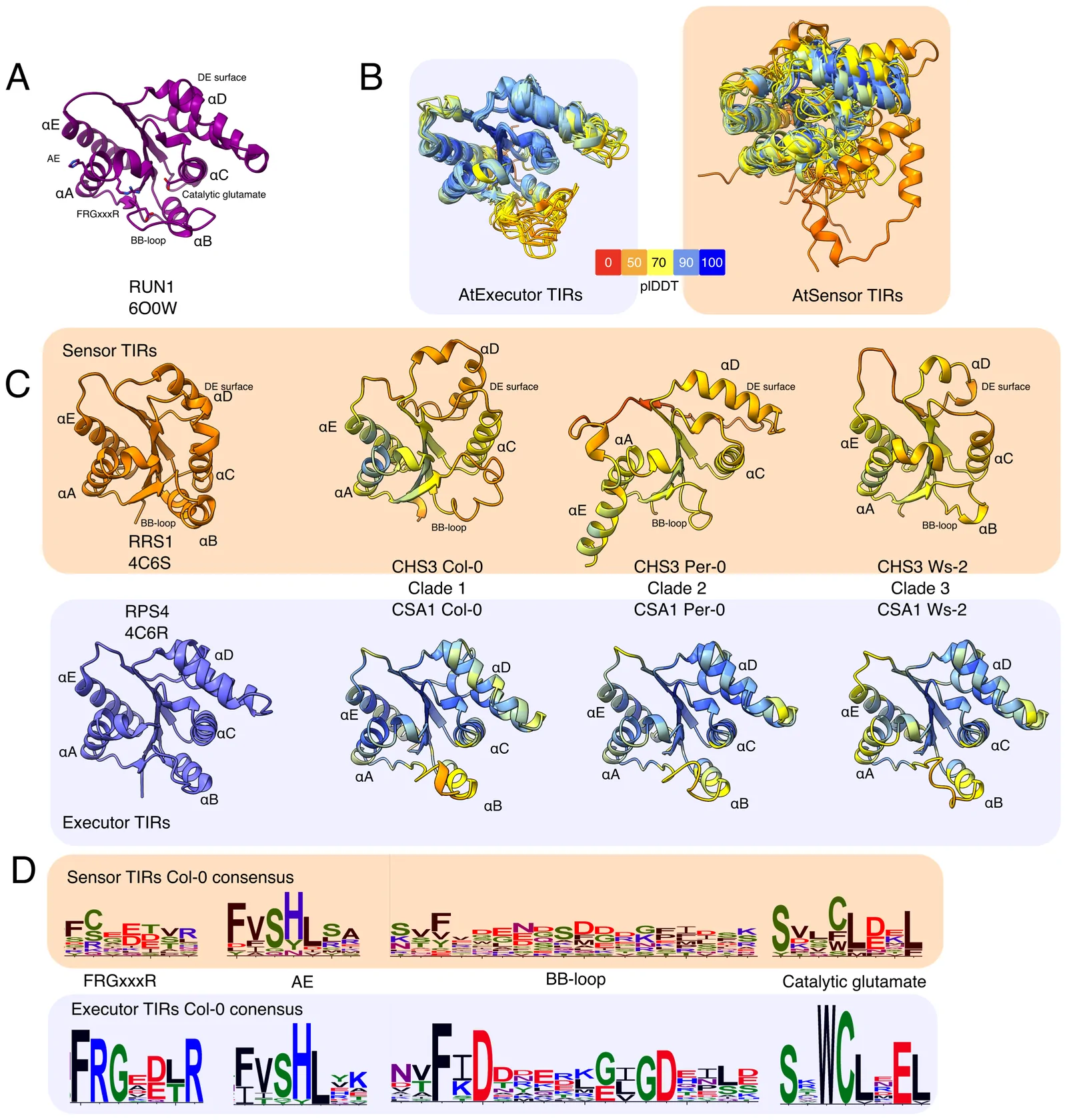
TIR domains of sensor NLRs vary significantly. **(A)** an example of a canonical enzymatic TIR domain from the singleton TNL *Muscadinia rotundifolia* RUN1 (PDB ID 6O0W). The alpha helices for which the key motifs are named are labelled. Important motifs in the function of enzymatic TIRs are highlighted, including the FRGxxxR motif, the AE interface (AE), BB-loop, Catalytic glutamate and the DE surface. **(B)** Alphafold 3 predicted structures of the TIR domains from each of the executor and sensor NLRs in **Figure 1**. Colouring by plDDT, where a score above 90 represents a confident prediction, and below 70 a low confidence prediction. **(C)** the Alphafold 3 predicted structures of CHS3 and CSA1 clades, showing sensor TIR variability between accessions of the same gene. RRS1 (PDB ID 4C6S) and RPS4 (PDB ID 4C6R) experimentally derived structures shown for comparison. **(D)** Web logo of the consensus sequences of sensor and executor NLRs from *Arabidopsis thaliana* Col-0 for key motifs involved in TIR signalling (Crooks et al., 2004).

TIR domains of sensor NLRs have diverged from each other, suggestive of genetic drift (**Figure 2B**). For example, in some sensor TIRs, the BB-loops are shorter (RRS1) and more variable in sequence compared to the executors (CHS3, CHL1), or missing completely (Induced by *Serendipita indica*, ISI) (Ahn et al., 2025). The catalytic glutamate is present in some sensors (AT3G51560, AT2G17050, CHL1, CHS3) but for CHS1, presence of catalytic glutamate is not coincident with an intact BB-loop or FRGxxxR motif, or the ‘SH’ motif in the AE interface (Williams et al., 2014). The DE surface region, forming the other half of the BE interface (Zhang et al., 2017a), is highly variable in sensor pairs, with some missing the characteristic three helices, resembling a RRS1-type TIR domain, whilst others have elongated αD helices relative to the executor pairs (**Figure 2B**), like the first TIR domain in SADR1-p (Jacob et al., 2023).

Even across accessions of the same sensor there is great variability (**Figure 2C**). Col-0 (Clade 1), Per-0 (Clade 2) and Ws-2 (Clade 3) accessions each represent one of the three clades of CHS3 (Yang et al., 2022, Yang et al., 2024) and show variability within their TIR domains. Differences in BB-loop length and composition, presence and absence of the catalytic glutamate region (WCLME motif) on the αC helix and large variation in the αD and αE helices are observed, the latter of which form a key part of the AE interface (**Figure 2C**). By contrast CSA1 (executor) TIRs are highly homogeneous (~65% identical residues compared to ~25% for CHS3 sensor pairs), all are identical in length and secondary structure, with variation only in and around the αD and αE helices (**Figure 2C**).

When looking at experimentally determined structures of RRS1 and model predictions of the sensor TIR domains, structural and sequence divergence mainly occurs around sites contributing to production of small molecule signalling metabolites (**Figures 2B, 2D**). Without the requirement for enzymatic activity, the sensor TIR domain has likely degenerated from the classical executor TIR and begun to adapt to fulfil a role within the executor-sensor pair, through interaction and stabilisation of the heteromeric complex (RPS4/RRS1), or through ID domains (RRS1, CHS3) and guardee (SOC3/CHS1/TN2) in other pairs.

### NB-ARC domains

The sensor pairs of the Arabidopsis NLRome segregate into two main subclades, the RRS1-like and the CHS1-like subclades (Van De Weyer et al., 2019). The CHS1-like clade consists of many TNs, which lack LRR domain as well as subdomains of the NB-ARC domain (Meyers et al., 2002). TN2 for example is missing both ARC1 and ARC2 subdomains, whilst CHS1 is missing the ARC2 subdomain (**Figure 1C**).

Like the TIR domain of sensors, there are also differing functional requirements of the NB-ARC domains in sensor NLR function. Each of the predicted pairs from Arabidopsis Col-0 accession have a conserved p-loop motif, however the requirement for function is variable across sensors, with RRS1 able to function without (Williams et al., 2014) but CHS3 and CHS1 requiring an intact p-loop for function (Liang et al., 2020; Yang et al., 2022, Yang et al., 2024).

The Walker B motif, involved in coordination of Mg^2+^ for ATP hydrolysis in NB-ARC containing proteins is largely well conserved across sensor NLRs, as is the sensor-1 motif, which recognises the (s) phosphate of ATP. Cryo-EM structural study of singleton TNL RPP1 delineate the sensor-1 motif into two subgroups, however both contain sensors and executor NLRs (Ma et al., 2020). The sequence variation of GLPL motif, a molecular hinge motif, and the MHD motifs, involved in the switch between resting and active NLR states, align with the NB-ARC domain clustering of TNL pairs (Van De Weyer et al., 2019). These motifs are better conserved in the executor pairs, but not the sensors.

ROQ1, RPP1, M and WRR4A form a tetrameric oligomer through their NB-ARC domains (Ma et al., 2020; Martin et al., 2020; Maruta et al., 2026a; Zhao et al., 2026). The residues in these NB-ARC oligomer interface residues are not conserved in sensor NLRs. This lack of conservation among predicted tetrameric resistosome interface residues suggests a different interface formation in sensors NLRs and highlights the distinct role of sensors in paired NLRs. Despite this lack of conservation there are no distinct sequence motifs within the NB-ARC domain that would allow identification of a paired sensor from singleton TNLs.

### LRRs and c-JIDs of pairs

Around 30% of putative sensor pairs, for example, CHS1, TN4, TN5, CHL1 and TN20 in Arabidopsis do not have LRR domain or other canonical C-terminal domains. For those that have LRR domains, there is significant variability in the number of repeats and some also lack the c-JID (RPP2A, ISI, STM1) (**Figure 1C**). Some singleton NLRs also lack c-JID, and therefore this is not unique to sensor NLRs (Johanndrees et al., 2023).

The sensor c-JID domains are overall less conserved than the executor c-JID domains (Saucet et al., 2021). RRS1, RPP2C, WRKY19, AT4G36140 and RRS1B have additional globular domain associated with their c-JID domains, not present in executor NLRs (Ahn et al., 2025). Overall, these c-JIDs, defined by four key motifs in Saucet et al. (2021), show significant variability across the sensor pairs, likely to do with their diverse functions within their pairs (Saucet et al., 2021), away from the role of effector binding and inhibition by ROQ1 c-JID (Martin et al., 2020). The c-JID in RRS1 is critical for RPS4 function and activation/autoinhibition (Sohn et al., 2014; Newman et al., 2019) and have evolved closely with the homologues from these pairs.

Identification of paired sensor NLRs is difficult, as they tend to be indistinguishable from executor/singleton NLRs in HMMER searches with the canonical domains TIR, NB, LRR (Kim et al., 2020; Steuernagel et al., 2020; Kourelis et al., 2021). Strategies for looking for IDs in NLRs has proven effective at identification of sensors and their neighbouring executor (Bailey et al., 2018), but we suggest careful inspection of the TIR sequences, and NB-ARC and c-JID domains should be considered alongside ID search. Sensor sequence divergence clearly points to a specialisation in the repression/detection of effectors within the context of its paired executor NLR, with specialisation occurring to such an extent that sensors of different clades within Arabidopsis genes (CSA1/CHS3) can no longer function together appropriately (Yang et al., 2022).

## Integrated domain of sensors and their effector recognition

Following work on the HMA domains in RGA4/RGA5 (Okuyama et al., 2011; Cesari et al., 2013), the integrated decoy model of NLR activation was proposed (Cesari et al., 2014). In contrast to the classical guard/decoy model, whereby the NLR senses changes in a host susceptibility target, the sensor NLR contains a domain mimicking this target, allowing for rapid recognition and detection of perturbations to the susceptibility factor. This binding or modification of the decoy domain/ID, then triggers the executor NLR. The diversity of sensor domains within the paired NLRs provide attractive targets for engineering through subtle modifications of existing IDs, or even the introduction of new targets and synthetic designed protein modifications (Kourelis et al., 2023; Xi et al., 2026; Zhu et al., 2026). Here we describe current knowledge on IDs of paired TNLs. We further explain interaction of IDs with cognate effectors through RPS4/RRS1, the only paired TNL with known cognate effectors to date.

Sensor NLRs can contain one or multiple IDs, typically these are domains other than canonical TIR/CC/NB-ARC/LRR/c-JID domains. Many of these domains bear resemblance to potential or predicted effector targets. The RRS1-R integrated WRKY domain is derived from major family of plant immune response regulators, and works as decoy for at least three different pathogen effectors (Zhang et al., 2017c; Mukhi et al., 2021; Lauber et al., 2024). The LIM-peptidase/DA1-like domains of CHS3/DAR4 resemble the DA1-like family of peptidases and are thought to be an effector target for *Albugo candida* (Gu et al., 2023). Other common IDs predicted to be found in paired TNLs include other transcription factor-like proteins, with folds including zinc-finger like domains, DNA binding domains, protein kinase domains and plant specific domains such as the calmodulin domains. Gene Ontology (Cesari et al., 2014; Kroj et al., 2016; Sarris et al., 2016) suggest these transcription factor-like domains to be involved in immune signalling and defence pathways. How these domains function in the effector/ligand recognition and if they might aid in repression/activation of the executor/sensor pair complex is of further interest.

## Domain-domain interactions of sensor and executor pair NLRs

Interaction between executor RPS4 and sensor RRS1-R are dependent on multiple domains. Structural studies of RPS4 and RRS1-R TIR domains identified the AE interface that mediates the heterodimerization of RPS4/RRS1-R (Williams et al., 2014), and RRS1-R AE interface mutants fail to oligomerise with RPS4 (Ahn et al., 2025). However, the BB-loop of RPS4 s not required for oligomerization (Ahn et al., 2025). A C15Y mutation of RRS1-R identified from mutant screen (Sohn et al., 2014) reduced association with RPS4 (Newman et al., 2019), suggesting various residues in RRS1-R TIR domain is required for interaction with RPS4. Whilst protein complex size is unaltered before and after effector recognition (Ahn et al., 2025), split-fluorescence assays with N-terminally tagged RPS4/RRS1-R reveal reduced proximity of RPS4 and RRS1-R upon activation (Guo et al., 2020). Further structural characterisation is required whether conformational rearrangements lead to steric hindrance of the fluorophores, or heterogeneous complexes exist that is indistinguishable on blue native-PAGE assays.

CSA1/CHS3 interaction is also dependent on the TIR domains. Clade 2 and 3 CSA1 and CHS3 requires intact AE interface of both TIR domains for interaction. However, whilst BB-loop of both CSA1 and CHS3 in clade 2 is not required for interaction, CHS3 BB-loop is required for CSA1/CHS3 interaction in clade 3 (Yang et al., 2024). SOC3/CHS1 pair is an interesting diversion, as interaction with CHS1 is dependent on the NB and LRR domain of SOC3, and not the TIR domain (Zhang et al., 2017b).

In the NB-ARC domain, the p-loop motif of both CSA1 and CHS3 are required for CSA1/CHS3 interaction (Yang et al., 2024). In contrast to CSA1/CHS3, only the p-loop motif of RPS4 is required for effector-dependent activation (Williams et al., 2014). Consistent with the phenotype, the p-loop mutant of RPS4 fails to oligomerise with RRS1-R, whilst corresponding RRS1-R mutant still oligomerises with RPS4 (Ahn et al., 2025). One of the mutants also identified through the mutant screen was C783Y in RPS4, located in the LRR domain (Sohn et al., 2014). This mutation fails to induce effector-dependent activation, and RPS4/RRS1-R fails to oligomerise, although protein stability of RPS4 was also significantly reduced (Ahn et al., 2025).

Intramolecular domain-domain interaction of RPS4/RRS1 is also essential for effector recognition. The c-JID domain (D4 in Ma et al., 2018) of RRS1 interacts with the WRKY domain and IDR (intrinsically disordered region) (previously D5, D6, respectively in Ma et al., 2018) in the absence of effector activation, but this interaction is reduced upon co-expression of AvrRps4 and PopP2 (Ma et al., 2018). This change in intramolecular interaction subsequently affects intermolecular interaction between RPS4 and RRS1. However, changes in interaction of RPS4 and RRS1 is dependent on the effector. Interaction between c-JID of RPS4 (previously CTD in Ma et al., 2018) and C-terminal domains (c-JID, WRKY, IDR) of RRS1-R is reduced upon AvrRps4 co-expression, but not PopP2 co-expression (Ma et al., 2018).

## Mechanism of autoimmunity, repression and derepression in Arabidopsis paired TNLs

There are about 15 pairs of paired TNLs identified in Arabidopsis Col-0, some studied more extensively than others. Three conserved mechanisms across these paired TNLs are observed; 1) autoimmunity mediated by executor misregulation, 2) repression of autoimmunity by the sensor, and 3) derepression by effector-dependent modification of the sensor. In this section, and in **Figure 3**, we give examples of the most extensively studied Arabidopsis paired TNLs and explain the conserved mechanism which maintains the delicate balance of preventing spontaneous activation, whilst preserving the ability to undergo rapid activation upon effector-dependent or independent activation.

**Figure 3:**
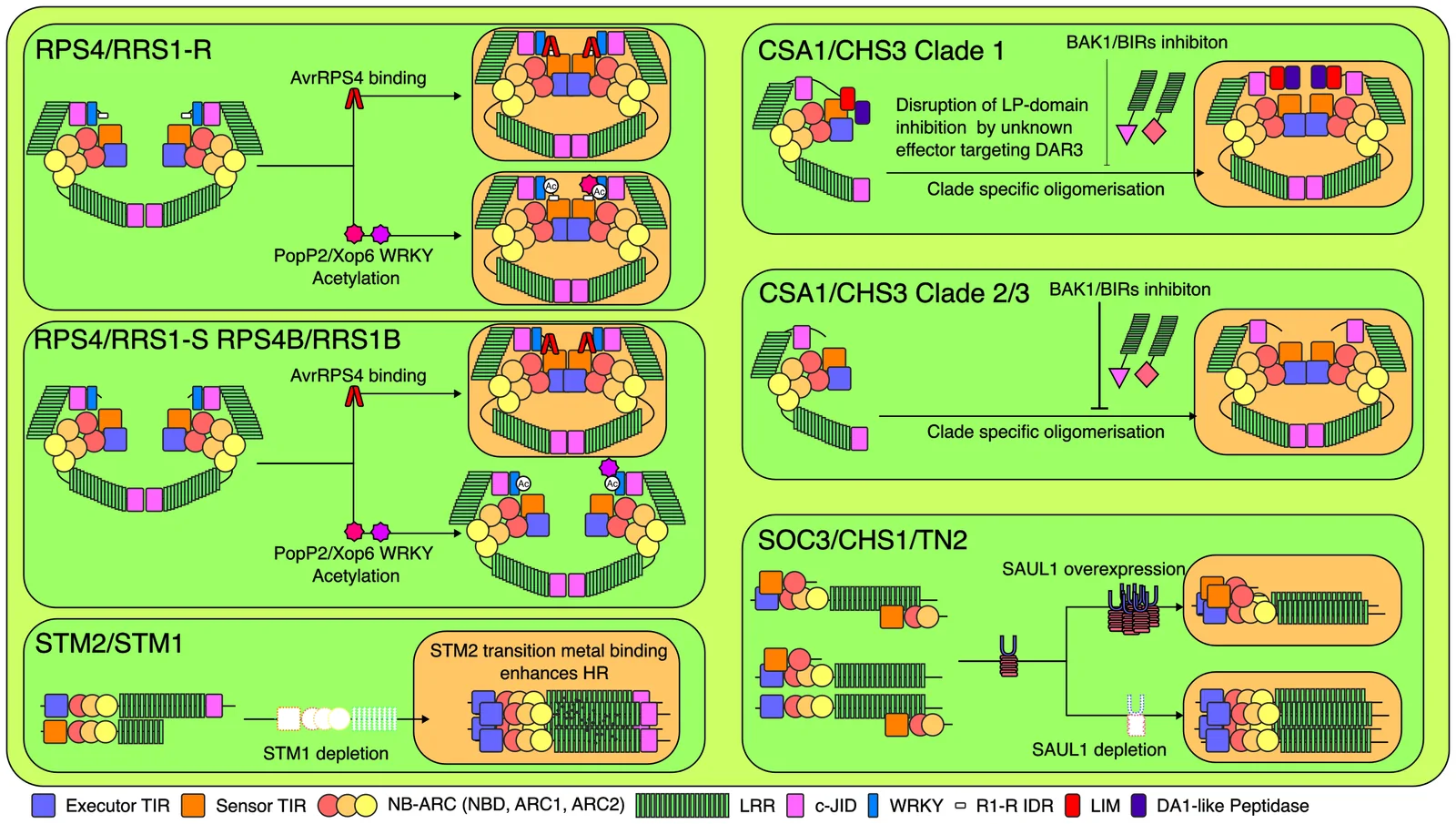
Mechanisms of paired NLRs from Arabidopsis. Paired NLRs have a diverse range of activation, repression and derepression mechanisms, of which key pairs are described here. Some alleles of RPS4/RRS1 are able to detect and respond to a range of effectors, specifically RRS1-R with its C-terminal IDR, allowing detection of PopP2, XopJ6 and an as yet unidentified effector from *Colletotrichum higginsianum*. RPS4-RRS1 exist in a preformed complex prior to effector recognition, with effector induced domain-domain interactions and conformational change though to activate the complex. STM2/STM1 balance transition metal binding and resistance to bacterial wilt. STM1 inhibits activity of STM2, however in *stm1* mutants, STM2 is able to cause HR, which is boosted in severity/intensity by the presence of transition metals. CSA1/CHS3 have numerous clades with differing activation requirements. Clade 1 CHS3 can act as a decoy for DAR3 through its integrated LIM-peptidase domains, and respond to depletion of BAK1/BIR proteins. Clades 2 and 3 are missing the integrated domain, but can still recognise BAK1/BIR protein modification by effectors. Clades 2 and 3 have distinct TIR domains, that require different activation requirements. The SOC3/CHS1/TN2 ‘pair’ is able to monitor the overexpression/increased activity of the SAUL1 E3 ligase, and the depletion of SAUL1, through SOC3/TN2 and SOC3/CHS1 complexes respectively. Whether these exist as pools of subcomplexes, or one large super complex is unknown. TIR, Toll/Interleukin-1 receptor; NB-ARC, Nucleotide Binding domain shared by APAF-1, apoptotic protease-activating factor 1; R proteins and CED-4 ,cell death protein 4; LRR, Leucine Rich Repeat; c-JID , C-terminal jelly roll/Ig-like domain; WRKY, WRKY transcription factor domain; R1-R IDR, RRS1-R Intrinsically Disordered Region; LIM, Lin-11, Isl-1, and Mec-3 domain.

### RPS4/RRS1 – RPS4B/RRS1B

RPS4 and RRS1-R or S from Arabidopsis Ws-2 and Col-0 accessions respectively, is the most extensively studied paired TNLs. RPS4 is a canonical TNL with TIR, NB-ARC, LRR and c-JID domains and acts as the executor of the pair. RRS1 contains WRKY domain which can bind DNA (Le Roux et al., 2015; Sarris et al., 2015). RPS4 pairs with RRS1 in either accession, as well as its duplicate pair RPS4B/RRS1B to provide resistance to *P. syringae* pv. *pisi* effector AvrRps4 (Narusaka et al., 2009; Sarris et al., 2015). The 83 amino acid extension at the C-terminus of RRS1-R allele from Ws-2 accession is required for detection of the *Ralstonia solanacearum* effector PopP2 (Hinsch and Staskawicz, 1996; Sohn et al., 2012), the *Xanthomonas campestris* pv. *campestris* effector XopJ6 (Deslandes et al., 2002) and the hemibiotrophic fungal pathogen *Colletotrichum higginsianum* (Birker et al., 2009; Narusaka et al., 2009).

Overexpression of RPS4 leads to autoimmunity in Arabidopsis (Wirthmueller et al., 2007) and localised cell death HR in *Nicotiana tabacum* (Zhang et al., 2004). A mutant uncovered in increased sensitivity to low humidity, *slh1* (sensitive to low humidity 1) was mapped to RRS1 with an additional leucine residue proximal to the WRKY domain (Noutoshi et al., 2005). Suppressor screening of *slh1* identified multiple mutations on RPS4 suggesting autoimmunity of *slh1* is derived from misregulation of RPS4 (Sohn et al., 2014).

Overexpression of RPS4 TIR domain in *N. tabacum* can be repressed by co-expression of RRS1 TIR domain (Williams et al., 2014). Similarly, overexpression of RRS1 mitigates the autoimmune phenotype following RPS4 overexpression (Huh et al., 2017). RPS4-mediated autoimmunity is also observed when the WRKY domain is replaced with another DNA binding domain such as LexA, or when domains C-terminal to the LRR domain in RRS1 has been deleted (Ma et al., 2018). RPS4 autoimmunity is observed through mutations in the WRKY domain (Noutoshi et al., 2005; Le Roux et al., 2015; Sarris et al., 2015; Guo et al., 2020). This suggests both TIR and WRKY domains of RRS1 are involved in repression of RPS4. Consistent with this intermolecular repression of RPS4 by RRS1, RRS1 and RPS4 form a complex in the absence of effectors, both in transient expression (Williams et al., 2014; Huh et al., 2017) and in native Arabidopsis (Ngou et al., 2020; Ahn et al., 2025).

In contrast, *rrs1–11* null allele overexpressing RPS4 showed mitigated autoimmunity compared to wild-type (Heidrich et al., 2013), suggesting a positive role for RRS1 in RPS4 autoimmunity. Furthermore, whilst RRS1-R repressed the autoimmunity in wild-type RPS4, RRS1-R potentiated the HR in RPS4 NB-ARC mutants S270F and D315E (Guo et al., 2021). The authors suggest these mutants could represent intermediate states and that upon activation of the complex, RRS1-R moves from a negative to a positive regulatory role.

RRS1 ‘senses’ pathogen effectors PopP2 (and likely XopJ6) and AvrRps4 via its WRKY domain through two different mechanisms. PopP2 is an acetyltransferase (Le Roux et al., 2015; Sarris et al., 2015) able to acetylate multiple host WRKY transcription factors, including the WRKY domain of RRS1. This acetylation disrupts RRS1-R binding to the W box DNA sequence (Le Roux et al., 2015; Sarris et al., 2015; Zhang et al., 2017c) leading to activation of the RPS4-RRS1 complex. Alongside the WRKY, a C-terminal extension present in RRS1-R but absent in RRS1-S, is required for full RPS4-RRS1 defence activation upon PopP2 recognition, but not AvrRps4, with PopP2 altering interaction between domains of the RRS1-RPS4 complex to activate the complex (Sarris et al., 2015; Ma et al., 2018; Guo et al., 2020). XopJ6, an effector from the bacteria *Xanthomonas campestris*, is from the same acetyltransferase family as PopP2 and also targets RRS1 WRKY domain for acetylation (Lauber et al., 2024). A natural variant of XopJ6 that disrupts binding to WRKY domain evades RRS1-RPS4 recognition (Lauber et al., 2024).

Similarly to PopP2 and XopJ6, AvrRps4 targets RRS1 WRKY. AvrRps4 binding also disrupts WRKY interaction with W box DNA (Mukhi et al., 2021). Acetylation of WRKY domain by PopP2 disrupts AvrRps4-WRKY interaction (Sarris et al., 2015). The crystal structure of the RRS1 WRKY with AvrRps4 (Mukhi et al., 2021) and PopP2 (Zhang et al., 2017c) further show that the two effectors bind to the same site of the WRKY domain. The binding of the effectors is necessary but not sufficient for RPS4/RRS1 activation (Sarris et al., 2015; Ma et al., 2018; Mukhi et al., 2021). Overall, the integrated WRKY domain of RRS1 is the target for multiple effectors by mimicking the pathogen targets (Le Roux et al., 2015; Sarris et al., 2015; Lauber et al., 2024) and the recognition of the effector is involved in the release of sensor repression on the paired executor NLR. Upon recognition, conformational change of RRS1/RPS4 complex is induced without a change in stoichiometry, which induces NADase activity, possibly by rearrangement of TIR domains (Ahn et al., 2025).

Post-translational modifications are also involved in derepression of the RPS4/RRS1 complex. WRKY domain and the following IDR of RRS1-R, but not RRS1-S is phosphorylated (Guo et al., 2020). Phosphorylation of serine residues in the IDR are required for PopP2 recognition, and phosphomimetic mutants are autoimmune in the absence of effector. In contrast, loss of T1214 phosphorylation leads to autoimmunity (Guo et al., 2020). RRS1 WRKY domain is also ubiquitinated by E3 ligase RARE (Chen et al., 2025). RARE destabilizes the RPS4/RRS1 complex, whilst deubiquitination enzyme UBP12/UBP13 removes ubiquitination by RARE (Chen et al., 2025). RPS4B is phosphorylated by BIK1 (Botrytis Induced Kinase 1), a major regulator of cell surface immunity. RPS4B S520A phosphorylation dampens AvrRps4-dependent activation (Toth et al., 2026), suggesting multiple regulatory layers of RPS4 or RPS4B derepression in addition to effector-dependent activation.

### CSA1/CHS3: clades with diverse function

CSA1 and CHS3 are both required for resistance to the oomycete *A. candida*. The autoactive mutant phenotype of *chs3-2D* is dependent on CSA1, and thus established CSA1/CHS3 as paired immune receptors (Xu et al., 2015). The *chs3-2D* autoactive allele contains a mutation in the LIM-peptidase domain, an integrated domain found at the C-terminal end of the LRR of CHS3. The LIM-peptidase domain is present in the DA1 family of growth regulators in Arabidopsis. LIM domains in DA1 proteins comprise a pair of zinc finger motifs for protein-protein interactions and a C-terminal peptidase domain with a diverse range of targets, DA1 proteins themselves are potential susceptibility/immunity factors.

CSA1/CHS3 is the most evolutionarily diverse sensor-executor pair in Arabidopsis (Yang et al., 2022), with three distinct clades (Van De Weyer et al., 2019). Clade 1 is distinguished by the presence of the LIM-peptidase domain, whilst clades 2 and 3 lack integrated domains. Clade 1 utilises the LIM-peptidase domain as both a guardee/decoy domain (Gu et al., 2023), and an immune suppression domain, whilst all three clades of CSA1/CHS3 guard, and are repressed by, BAK1 (BRASSINOSTEROID INSENSITIVE 1-ASSOCIATED KINASE 1) and BIR (BAK1-INTERACTING RECEPTOR-LIKE KINASE) proteins (Yang et al., 2010, Yang et al., 2022). Absence of the LIM-peptidase domain in clades 2 and 3 of CHS3 (Yang et al., 2022, Yang et al., 2024) point to a diversification of function/selection pressures that likely enabled evolution of the integrated domain version of CSA1/CSH3.

LIM-peptidase domains of other DA1 family members, such as DAR3 (DA1-related 3), complements CHS3 LIM-peptidase ID-dependent HR, suggestive of shared features between them which maintain an inactive CSA1/CHS3 complex (Gu et al., 2023) further strengthening the identity of the LIM-peptidase domain as a decoy domain. Integrity of the LIM-peptidase domain is therefore the point of regulation of the CSA1/CHS3 clade 1 pair. Accordingly, *chs3-2d* CSA1-dependent autoimmunity is inhibited by *in trans* overexpression of the LIM-peptidase domain of CHS3 (Yang et al., 2010). Presumably, as a decoy domain, the putative effector would interact, modify or destabilise the LIM-peptidase domain, directly or through host protein interaction (Gu et al., 2023) releasing inhibition of CSA1, and allowing formation of an oligomeric complex of unknown size with CHS3 (Yang et al., 2024).

In an alternative functional role, CSA1/CHS3 activation through oligomerisation is inhibited by the presence of BAK1/BIR3 (Schulze et al., 2022; Yang et al., 2024), in this case with CSA1 forming interactions with BIR3 and BAK1. CSA1/CHS3 clades 2 and 3 show strong HR when overexpressed, which can be repressed upon co-expression with BAK1 and BIR proteins. This repression is dose dependent, with increasing concentrations of BAK1 and/or BIR domains suppressing the formation of the active hetero-tetramer of clade 2/3 CS1/CHS3 protein complexes (Yang et al., 2024).

The evidence for clade 1 repression by BAK1 absence is more complex. The *chs3-2d* phenotype is recessive, as heterozygous expression of the *chs3-2d* mutant does not give the autoactive phenotype seen in homozygous lines (Yang et al., 2022). However, in *bak1* lines heterozygous mutants begin to show signs of dwarf phenotype, demonstrating a potential dose dependent repression of CSA1 clade 1 (Yang et al., 2022).

Upon pathogen modulation of the BAK1/BIR protein complexes, including *P. syringae* pv. *tomato* DC3000 *(Pto*) effector HopB1 cleavage of BAK1 (Schulze et al., 2022), CSA1/CHS3 oligomerise, forming a putative tetramer (Yang et al., 2024). The stoichiometry of the clade 2 and 3 active complexes appears to resemble that of RPS4/RRS1 (Ahn et al., 2025), forming complexes on BN-PAGE consistent with tetrameric assembly (Yang et al., 2024). CSA1/CHS3 clades 2 and 3 also appear to have different functional requirements of their TIR domains, likely due to the presence and absence of key TIR domain motifs (**Figure 2**) in a further divergence of function within the pair. CSA1/CHS3 demonstrate the diverse mechanistic activation and derepression mechanisms present in sensor-executor pairs through evolutionary ID-integration and may make a useful case study for integration of engineered or selected integrated domains into existing NLR pairs.

### SOC3/CHS1/TN2: pair plus one?

CHS1 (also known as TN1) was identified as *chs1*, a mutant with a chilling sensitive phenotype, despite growing normally at 23 °C (Schneider et al., 1995; Wang et al., 2013). SOC3 was identified as a suppressor of this autoactive mutant. TN2, positioned next to CHS1 on the chromosome, was also identified in another suppressor screen of *exo70b1* (Zhao et al., 2015; Liu et al., 2017).

The SOC3-CHS1-TN2 complexes function as guards of at least two different susceptibility factors in Arabidopsis. SOC3/CHS1 monitors the depletion of SAUL1 (Senescence-associated E3 ubiquitin ligase 1), an E3 ligase involved in immune responses (Tong et al., 2017). TN2 causes autoimmunity upon the overaccumulation of SAUL1, with TN2 thought to interact with targets of SAUL1 (Liang et al., 2019). Loss or overaccumulation of SAUL1 is sensed by SOC3/CHS1 and SOC3/TN2 respectively, leading to SOC3 dependent cell-death and immune responses. The *saul1–1* autoimmune phenotype requires the presence of CHS1 (Liang et al., 2019). SOC3/CHS1 and SOC3/TN2 complexes exist concurrently, as the two complexes do not compete for binding (Liang et al., 2019). Whether they exist as a super complex or as distinct pools is not yet apparent.

An alanine to threonine (A10T) mutation in the N-terminal IDR preceding the TIR domain of CHS1, *chs1-2* (Schneider et al., 1995), enables SOC3 dependent autoimmunity. Expression of the wild type CHS1 TIR and NB domains in the *chs1–2* background can supress this phenotype, via direct interaction through the NB and LRR domains of SOC3. The role of the derepression in the *chs1–2* mutation in the TIR domain is not clear, as it lies outside known interaction interfaces observed in other plants, and so may be involved in other intermolecular interactions within the SOC3/CHS1 complex. Additional mutations to the CHS1 TIR suppress the *chs1–2* autoactive phenotype (G152R and A138V), again demonstrating the need for a functional CHS1 for guard function.

TN2 has a second function as an indirect guard for the exocyst protein EXO70B1. Autoimmunity seen in the mutant *exo70b1* phenotype is not dependent on SOC3 or its homolog SOC3-L1 (Wang et al., 2020), but instead on the integrity of the TN2 TIR and NB domains (Zhao et al., 2015). This complex multi-guard system highlights the flexibility of paired complexes, able to recognise different modifications with different guards. This is interesting, as a sensor NLR, TN2 is still able to induce cell death when fused to tandem SAM domains (Bayless et al., 2025), but is unable to produce metabolites associated with the EDS1/SAG101/NRG1 cell death. The phenotypes associated with the SAUL1 overaccumulation, dependent on TN2, and the SAUL1 depletion, dependent on CHS1, are markedly different, suggestive of differential signalling through SAG101/NRG1 in case of SOC3 and CHS1, and PAD4/ADR1 pathways in case of TN2.

### DSC1/WRKY19: multiple putative ID

DSC1 (DOMINANT SUPRESSOR OF CAMTA3 NUMBER 1) was originally identified from suppressor screen of *camta3* mutants expressing dominant-negative NLR mutants with mutated p-loops (Lolle et al., 2017). However, a double mutant of *dsc1 camta3* was indistinguishable from *camta3* single mutant, and indeed, DSC2, another TIR-NLR was identified in *camta3* autoimmunity (Lolle et al., 2017). DSC2 is not paired with DSC1, but both are required to suppress *camta3* autoimmunity (Lolle et al., 2017).

DSC1 is located in head-to-head orientation with another TNL, WRKY19. In addition to the genomic location, TIR domain sequence of WRKY19 diverged like those of RRS1-like sensor paired NLRs. Warmerdam et al. (2020) reported that both DSC1 and WRKY19 may be required for conferring resistance against *Meloidogyne incognita* (root knot nematode) (Warmerdam et al., 2020). WRKY19 also has multiple IDs, including a N-terminal PAH (Paired Amphipathic Helix) domain (present in SIN3 and SIN3-like proteins, involved in transcriptional control) (Bowen et al., 2010), two WRKY domains preceding the TIR domain (Rushton et al., 2010) and a MAPK domain, highly similar to AtMEKK1 at the C-terminus (Warmerdam et al., 2020). Currently the functional requirements of these domains are unclear, but WRKY19 would make an attractive target for engineering of paired TNLs, with many IDs available to swap and manipulate to broaden host specificity.

### STM1/STM2 metal binding and immunity

The NLR pair STM1 and STM2, characterised by Gao et al. (2026) and found in the same NLR cluster as RPS4/RRS1, have a unique derepression model, involving a trade-off between resistance to *R. solanacearum* wilt and sensitivity to transition metals (Gao et al., 2026). In resting state, STM1 inhibits STM2 autoactivity. When STM1 is non-functional, STM2 is derepressed and able to form the NADase holoenzyme. Both STM1 and STM2 form homo- and hetero-oligomers, and STM2 autoimmunity is likely driven by its self-association, which is inhibited in the presence of STM1. In the absence of STM1 repression, STM2 binds transition metals, such as Cd^2+^, likely via the LRR domain which increases NADase activity and cell death strength via the EDS1/PAD4/ADR1 signalling pathway (Gao et al., 2026) and resistance to *R. solanacearum* (Demirjian et al., 2023; Gao et al., 2026). The authors speculate that transition metal binding may weaken autoinhibitory interactions with the LRR domain and TIR domains or affect interaction with an as yet unidentified effector. How this unique derepression mechanism functions mechanistically is of great interest.

## Evolving pairs

NLRs undergo duplication and often exist as clusters in the genome (Barragan and Weigel, 2021). For example, the RPP4/5 cluster is highly varied across Arabidopsis natural accessions (Teasdale et al., 2025). Unlike RPP1, most paired TNLs are conserved across the studied natural accessions (Van De Weyer et al., 2019), and are considered ancient (Lee and Chae, 2020). In contrast to the assumption that sensor paired NLRs would evolve faster than their counterpart executors, both sensor and executor show a co-evolution pattern, exhibiting high correlation of the Tajima’s D value (Van De Weyer et al., 2019). This explains the exclusive requirement of the sensor-executor pairs for function described above.

Recent studies identified non-typical paired TNLs, that function together with more than one gene, or function with genes that are not juxtaposed in the genome. Some of SOC3 function requires another TN, TN2, for function (Liang et al., 2019). CHS1 and TN2 are neighbouring in the genome, with high sequence similarity, suggesting a duplication event. CHS1 and TN2 can function redundantly with SOC3 (Liang et al., 2019). However, TN2 protein level is regulated by SAUL1, whereas CHS1 level and interaction with SOC3 is unaffected, indicating the neo-functionalization of the duplicated genes.

Kim et al. (2024) reported that four TNLs RPP2A/B/C/D are required for recognition of effector ATR2 and resistance against *Hpa* (Kim et al., 2024). RPP2A and RPP2B are the first pair NLR to be identified (Sinapidou et al., 2004), which does not have a shared promoter and are tandemly oriented. RPP2C and RPP2D on the other hand have shared promoter, and carry features of TIR domain sequence variation found in sensor paired TNLs (see above).

Whilst all the above examples are juxtaposed in the genome, another example of cooperative function of TNLs is with distantly positioned genes in the genome. Autoimmune phenotype of *snc1–1* is relieved by mutation of proximal genes SIKICs (SIDEKICK SNC1) (Dong et al., 2018). The three genes SIKIC1, SIKIC2 and SIKIC3 are tandemly oriented with high sequence similarity. The SIKICs function redundantly in repressing *snc1–1* autoimmunity, and interaction between SIKIC2 and SNC1 has been confirmed (Dong et al., 2018). Similarly, expression of CHS3 alone did not trigger cell death, but co-expression with TNT1 (AT3G04220) led to cell death (Lüdke et al., 2025). CHS3 and TNT1 are both located on Arabidopsis chromosome 5 but are distally located. Finally, the TIR domain of TNL40 and TNL60, which are paired with TN4 and TN5, respectively, only interact with TN10 TIR domain, and not with TIR domains of TN4 or TN5 (Chen et al., 2021). All these examples illustrate the possibility of cooperation between TNLs that are not pairs and distantly located in the chromosome, possibly a form of paired TNLs evolved due to gene duplication,

Recently, role of TIR domain-containing genes beyond effector recognition has been reported. In one report, TIR domain-containing genes were significantly overexpressed in vasculature upon infection (Tang et al., 2023). In a mutant screen, TNL SADR1 (AT4G36140) was identified to repress the autoimmune phenotype of the RNL mutant *adr1-l2* (Jacob et al., 2023). Although there is SADR1p (AT4G36150) in a head-to-head orientation with SADR1, the role of SADR1 downstream of *adr1-l2* does not require SADR1p (Jacob et al., 2023). Furthermore, Fliegmann et al. (2026) reported the requirement of TIR domain function in PTI response (Fliegmann et al., 2026). All these suggest the possibility of signalling cascade of TIR domain proteins that boost immune function. In this view, it may be possible that the correlated function of TNLs may be as a signalling cascade, rather than as paired TNLs. For these, constitutive interaction of different TNLs and existence of heteromeric TNL oligomers will be important to identify whether these TNLs function as variation of pair or as cascade.

## Distribution in other species

Consistent with the conservation of paired TNLs across Arabidopsis accessions, homologs of paired TNLs are identified in other Brassicaceae species. Lee and Chae (2020) identified paired TNL homologs in *Arabidopsis lyrata* (Lee and Chae, 2020). Interestingly, the TTR1 (Tolerance to Tobacco Ringspot virus 1) clade (RPS4B/RRS1B of Arabidopsis) is strictly duplicated in 1:1 ratio across all of *A. lyrata* accessions (Lee and Chae, 2020), consistent with the exclusive requirement of the sensor-executor pair for repression of autoimmunity. Other studies also identified RPS4/RRS1 orthologues from other Brassicaceae species. However, the conserved WRKY integrated domain of RRS1 is only apparent in RRS1 orthologues of Camelineae, which includes *Camelina sativa* and *Capsella rubella* (Saucet et al., 2015; Chen et al., 2025). RRS1 orthologues in other species of the Brassicaceae, such as *A. lyrata* and *Brassica rapa*, lack WRKY domain (Chen et al., 2025).

An RPS4/RRS1-like pair has been identified in *B. napus*. BnRPR1 (*Brassica napus* Resistance Paired Receptor 1) is similar in sequence to RRS1 and carries two integrated domains, a B3 domain that precedes the TIR domain, and a TFIISN (Transcription factor IIS N-terminal domain) after the LRR domain. Co-expression of BnRPR1 and its neighbouring gene BnRPR2 in heterologous *N. benthamiana* and *N. tabacum* leads to HR (Mermigka et al., 2023), similar to CSA1/CHS3 overexpression in *Nicotiana* species (Yang et al., 2022). Whilst it is possible to conclude cooperative function of BnRPR1/BnRPR2 pair, it is also possible that the pair requires a guardee specific to *B. napus*, and that HR is observed in the *Nicotiana* species is due to lack of this guardee, similar to observations in CSA1/CHS3 (Schulze et al., 2022; Yang et al., 2022).

Most paired TNLs are found in Brassicaceae species, but there is also an example from Cucurbitaceae. The Fom-1/Prv1 pair in melon confers resistance to *Fusarium oxysporum* Races 0 and 2 and to PRSV (Papaya ring-spot virus) (Brotman et al., 2013). The second NB domain of Prv interacts with PRSV cylindrical inclusion protein, suggesting the second NB domain may act as an integrated domain (Normantovich et al., 2024). Fom-1 contains canonical TNL domain architecture, and mutation of Fom-1 leads to loss of disease resistance. A mutation of Prv1 that generates a frameshift downstream of the TIR domain leads to autoimmune symptoms, suggesting mutant Prv1 fails to repress Fom-1, and is consistent with Prv1 function as sensor and Fom-1 as executor (Nizan et al., 2023; Muthusamy et al., 2026).

Identification of paired TNLs in Brassicaceae as well as in Cucurbitaceae suggest possibility of wide distribution of paired TNLs across rosids. The identification of autoimmune symptoms of BnRPR1/BnRPR2 and Fom-1/Prv1 upon transient expression in *Nicotiana benthamiana* further suggest conserved repression-derepression mechanism of paired TNLs across species (Mermigka et al., 2023). Transfer of both RPS4/RRS1 into asterid *N. benthamiana* successfully conferred HR to AvrRps4 and PopP2 expression (Narusaka et al., 2013). Future studies on the activation mechanism will therefore have applications across different crops species.

## Conclusion and future questions

Paired NLRs were identified over two decades ago. Since then, a great diversity of paired NLRs have been characterised consisting of both TNLs and CNLs. Here, we discuss identified and characterised paired TNLs with focus on Arabidopsis. More paired TNLs continue to be identified from different species, such as in cucurbits. We suggest that the head-to-head genomic structure and the sequence divergence of TIR domains can be used as a proxy for identification of more paired TNLs, coupled with biochemical data for direct protein-protein interaction, and when present, a characterisation of their IDs (**Figure 4**). We also outline three conserved mechanisms across paired TNLs; Autoimmunity by executor, repression of executor by sensor and derepression of executor by effector. Despite the advances, many questions remain in understanding paired TNLs. We highlight the following future questions for further study.

**Figure 4:**
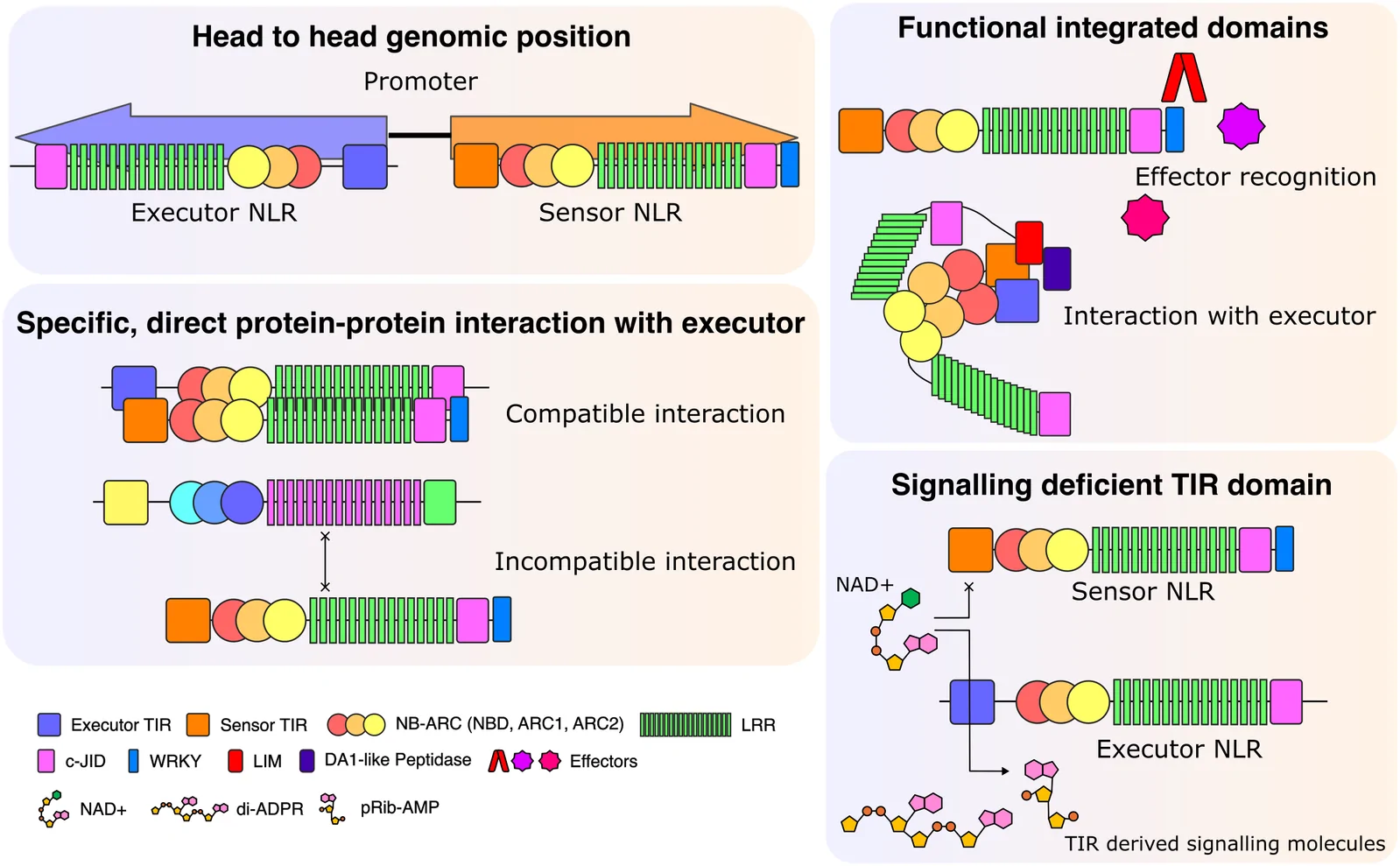
How to spot a sensor NLR. Most paired NLRs exist in a head-to-head genomic position, with a shared promoter and are divergently transcribed. Many, but not all sensor NLRs contain an integrated, non-canonical NLR domain for effector recognition, or interaction with the executor NLR. Sensor pairs also have an exclusive, specific, constitutive protein-protein interaction with the executor pair, often requiring one another for stability and repression of auto-activity. Finally, in TNL pairs, the sensor will likely have a TIR domain that is compromised in immune signalling, generally through loss of key interaction interfaces, or motifs involved in the production of NAD^+^-derived signalling molecules. Executor TIR domains hydrolyse the nicotinamide moiety from NAD^+^ and produce numerous signalling molecules that bind to EDS1/PAD4, or EDS1/SAG101 complexes. TIR, Toll/Interleukin-1 receptor; NB-ARC, Nucleotide Binding domain shared by APAF-1, apoptotic protease-activating factor 1, R proteins and CED-4, cell death protein 4; LRR, Leucine Rich Repeat; c-JID, C-terminal jelly roll/Ig-like domain; WRKY, WRKY transcription factor domain; LIM, Lin-11, Isl-1, and Mec-3 domain; NAD+ , Nicotinamide adenine dinucleotide, di-ADPR, Di-adenosine diphosphate ribose pRib-AMP, phospho-ribose-adenosine monophosphate.

- Molecular mechanism of paired TNL activation
- Regulation of paired TNLs to prevent autoimmunity
- Engineering of paired TNLs to recognise multiple effectors and/or pathogens, and thus achieving broad spectrum resistance without need for incorporation of multiple NLRs
- Variation of paired TNLs or TNL cascade in response to pathogen infection
